# Comparison of the effects on facial soft tissues produced by rapid and slow maxillary expansion using stereophotogrammetry: a randomized clinical trial

**DOI:** 10.1186/s40510-023-00498-9

**Published:** 2024-01-03

**Authors:** Matilde Marino Merlo, Bernardo Quiroga Souki, Michele Nieri, Agnese Bonanno, Veronica Giuntini, James A. McNamara Jr., Lorenzo Franchi

**Affiliations:** 1 Graduate Orthodontic Program, Department of Experimental and Clinical Medicine, The University of Florence, Florence, Italy; 2 Graduate Orthodontic Program, Pontifical Catholic University of Minas Gerais, Belo Horizonte, Brazil; 3 Department of Experimental and Clinical Medicine, Orthodontics, The University of Florence, Florence, Italy; 4 Thomas M. and Doris Graber Endowed Professor of Dentistry Emeritus, Department of Orthodontics and Pediatric Dentistry, School of Dentistry, The University of Michigan, Ann Arbor, MI USA; 5 Professor Emeritus of Cell and Developmental Biology, School of Medicine, The University of Michigan, Ann Arbor, MI USA; 6 Center for Human Growth and Development, The University of Michigan, Ann Arbor, MI USA; 7 Thomas M. Graber Visiting Scholar, Department of Orthodontics and Pediatric Dentistry, School of Dentistry, The University of Michigan, Ann Arbor, USA; 8 Director of the Division of Dentistry, University Hospital of Careggi, Via del Ponte di Mezzo, 46-48, 50127 Florence, Italy

## Abstract

**Objective:**

To compare the effects on facial soft tissues produced by maxillary expansion generated by rapid maxillary expansion (RME) versus slow maxillary expansion (SME).

**Materials and methods:**

Patients in the mixed dentition were included with a transverse discrepancy between the two arches of at least 3 mm. A conventional RME screw was compared to a new expansion screw (Leaf expander) designed to produce SME. Both screws were incorporated in a fixed expander. The primary outcome was the difference of the facial tissue changes in the nasal area measured on facial 3D images captured immediately before application of the expander (T0) and after one year of retention, immediately after the expander removal (T1). Secondary outcomes were soft tissue changes of other facial regions (mouth, lips, and chin). Analysis of covariance was used for statistical analysis.

**Results:**

Fourteen patients were allocated to the RME group, and 14 patients were allocated to the SME group. There were no dropouts. Nasal width change showed a difference between the two groups (1.3 mm greater in the RME group, 95% CI from 0.4 to 2.2, *P* = 0.005). Also, intercanthal width showed a difference between treatments (0.7 mm greater in the RME group, 95% CI from 0.0 to 1.3, *P* = 0.044). Nasal columella width, mouth width, nasal tip angle, upper lip angle, and lower lip angle did not show any statistically significant differences. The *Y*-axis (anterior–posterior) components of the nasal landmark showed a statistically significant difference between the two groups (0.5 mm of forward displacement greater in the RME group, 95% CI from 0.0 to 1.2, *P* = 0.040). Also, *Z*-axis (superior-inferior) components of the lower lip landmark was statistically significant (0.9 mm of downward displacement in favor of the RME group, 95% CI from 0.1 to 1.7, *P* = 0.027). All the other comparisons of the three-dimensional assessments were not statistically significant.

**Conclusions:**

RME produced significant facial soft tissue changes when compared to SME. RME induced greater increases in both nasal and intercanthal widths (1.3 mm and 0.7 mm, respectively). These findings, though statistically significant, probably are not clinically relevant.

*Trial registration* ISRCTN, ISRCTN18263886. Registered 8 November 2016, https://www.isrctn.com/ISRCTN18263886?q=Franchi&filters=&sort=&offset=2&totalResults=2&page=1&pageSize=10

**Supplementary Information:**

The online version contains supplementary material available at 10.1186/s40510-023-00498-9.

## Introduction

Rapid maxillary expansion (RME) is a common orthopedic procedure for the treatment of maxillary transverse deficiency [1–5]. RME is indicated in the treatment of orthodontic problems ranging from posterior crossbite to increasing available arch perimeter in mild-to-moderate crowding cases and sleep-disordered breathing [1, 5, 6]. Orthopedic maxillary expansion occurs in growing subjects with immature skeletal development when the force applied to the teeth, and the maxilla exceeds the limit needed for tooth movement [3]. Therefore, early treatment of maxillary transverse deficiency through palatal expansion is strongly recommended [1–5].

Maxillary expansion can be achieved using either fixed or removable appliances, and expansion can be either rapid or slow [2, 5]. RME is associated with systems producing heavy and intermittent forces applied in a short time frame and is achieved typically through fixed expanders anchored to teeth or tissues. On the contrary, slow maxillary expansion (SME) typically utilizes continuous low-force systems applied over a longer period, and it is achieved through removable or fixed expanders (e.g., Quad Helix appliance, removable plates, RME devices with slow activation protocols) [5, 7]. More recently, an increasing interest of researchers addresses toward fixed devices that are equipped with a screw whose activation generates the compression of two or more nickel titanium leaf springs that recover their original shape during deactivation (Leaf expander) [8–11].

One of the major concerns regarding the expansion procedure is the possible negative effects on the appearance of the face, with particular emphasis not only on the teeth, but also on the hard and soft tissues of the nose [12, 13]. Facial soft tissues are affected by expansion procedures as a result of changes in dentition and/or in skeletal structures [12–14]. Huang et al. [12] concluded that RME can produce significant increases in nasal width, mouth width, upper philtrum width, and distance from the lower lip to the E-line after the retention phase. However, the clinical relevance of these findings remains questionable.

Technological advances in recent years have enabled orthodontists and maxillofacial surgeons to use the 3D facial scanning systems like laser surface scanning and stereophotogrammetry. These methods are fast, not invasive and allows evaluation of facial structures without exposing the patient to radiation [12–15]. Inclusion of surface texture is another advantage of the system [16].

Despite the increasing interest on the effects produced by SME, to our knowledge, no previous study compared the effects on facial soft tissues produced by RME and SME using stereophotogrammetry. In particular, it would be useful to know if these two techniques may result in significant nasal changes. Therefore, the objective of this RCT was to compare the effects on facial soft tissues produced by maxillary expansion generated by the conventional RME screw versus the Leaf expander screw through digital stereophotogrammetry. The null hypothesis was that soft tissue changes are not significantly different between RME and SME groups.

## Methods

The present RCT follows the guidelines of CONSORT 2010 [17].

### Trial design

This is a superiority, single-center, two arms parallel-balanced randomization trial. This study was conducted in the Orthodontic Clinic of the Careggi University Hospital, Florence, Italy, from October 2016 to November 2018. The study was registered in the ISRCTN register on 08/11/2016 with the ISRCTN18263886 number. Participants of this study had been enrolled in one of two centers where the equipment for digital stereophotogrammetry was available.

### Participants

To be included in the present study, patients had to present with a prepubertal phase of development (cervical stage [CS] 1 or 2 in cervical vertebral maturation [18]), and in the early or intermediate mixed dentition stage [19] with fully erupted maxillary and mandibular permanent first molars. Other inclusion criteria were the presence of the maxillary deciduous second molars available as anchoring teeth (the second deciduous molar was considered available as anchoring tooth when the root had the same length as the clinical crown at the radiographic examination, [20]) and a posterior transverse interarch discrepancy (PTID) [21] of at least  3 mm.

Exclusion criteria were the following: pubertal or postpubertal stage of development (CS 3–6), age older than 14 years, late deciduous or late mixed dentition, Class III malocclusion, congenitally missing maxillary second premolars, cleft lip and/or palate and craniofacial syndromes, and patients unable to be followed for at least 1 year.

All patient’s parents signed an informed consent before starting the trial. The study was approved by the Pediatric Ethics Committee of Tuscany, Italy (No. 57/2016).

### Interventions

In the RME group, maxillary expansion was performed with a butterfly expander. The appliance consists of a conventional RME screw (A2620—Leone SpA, Sesto Fiorentino, Firenze, Italy) with a butterfly-shaped stainless-steel framework that extends forward to the palatal surfaces of the maxillary first deciduous molars with two bands cemented on the maxillary second deciduous molars (Additional file 1: Fig. 1) [22].

In the SME group, maxillary expansion was carried out with an expansion screw that delivered continuous moderate forces (Leaf Expander—Leone SpA, Sesto Fiorentino, Firenze, Italy). Also, in this group, the expander had a butterfly design with bands cemented on maxillary second deciduous molars (Additional file 2: Fig. 2).

In both RME and SME groups, a 10-mm screw was used. If the 10-mm screw was not sufficient to correct the transverse interarch discrepancy, a second expansion phase was planned.

In the RME group, patients’ parents were instructed to activate the screw 1/4 turn per day (one activation corresponded to 0.2 mm per turn), immediately after the appliance was cemented in place. Patients were checked every 2 weeks when approximately 3 mm of expansion was obtained.

In the SME group, the NiTi screws of the Leaf expander developed 900 g of force. An initial expansion of 4.5 mm was achieved in about 2–3 months. Patients were checked every 2 weeks to monitor the deactivation of the spring. The expansion screw was activated monthly by 15-quarter turns. (One-quarter of turn corresponded to 0.1 mm of activation, thus 15 activations of the screw generated 1.5 mm of activation.)

In both groups, the expansion screw was activated until the desired palatal expansion was reached (palatal cusps of the maxillary second deciduous molars approximating contacting the buccal cusps of the mandibular second deciduous molars). All expanders remained in place as passive retainers and were removed one year after their initial cementation.

Three clinicians treated all the patients. The previous clinical experience was similar for all clinicians (5–10 years of clinical practice).

### Facial imaging with digital stereophotogrammetry

3D facial surface images of all patients were captured in natural head position using the Face Shape Maxi 3D Scanner (Polishape 3D srl, Bari, Italy). This scanner captured images by means of six cameras (Canon 1200D 18Mpx, Canon, Tokyo, Japan) and two external flashes (Metz BL-400 SB Kit, Metz Consumer Electronics GmbH, Zirndorf, Germany), synchronized and mutually arranged in a predetermined orientation. Each patient was positioned on an adjustable stool and instructed to look into his/her eyes in a mirror placed at 2 m distance, with teeth in occlusion, eyes open, and lips relaxed and in contact. Hair was pulled back to leave the forehead and ears uncovered. All the images were saved as.stl files with no standardized orientation in the Cartesian plane.

### Outcomes

The primary outcome of this study was the difference of the facial tissue changes in the nasal area between RME group and SME group, measured on facial 3D images captured immediately before application of the expander (T0) and after one year of retention, immediately after the expander removal (T1). Secondary outcomes were soft tissue changes of other facial regions (mouth, lips, and chin).

Distances of specific landmarks between T0 and T1 models were calculated with the following procedure. The T0 and T1 models were oriented first using the plane of Camper, the anthropometric plane extending from the tip of the anterior nasal spine (acanthion) to the superior border of the tragus of the ears on the right and left sides [23].

**Fig. 1:**
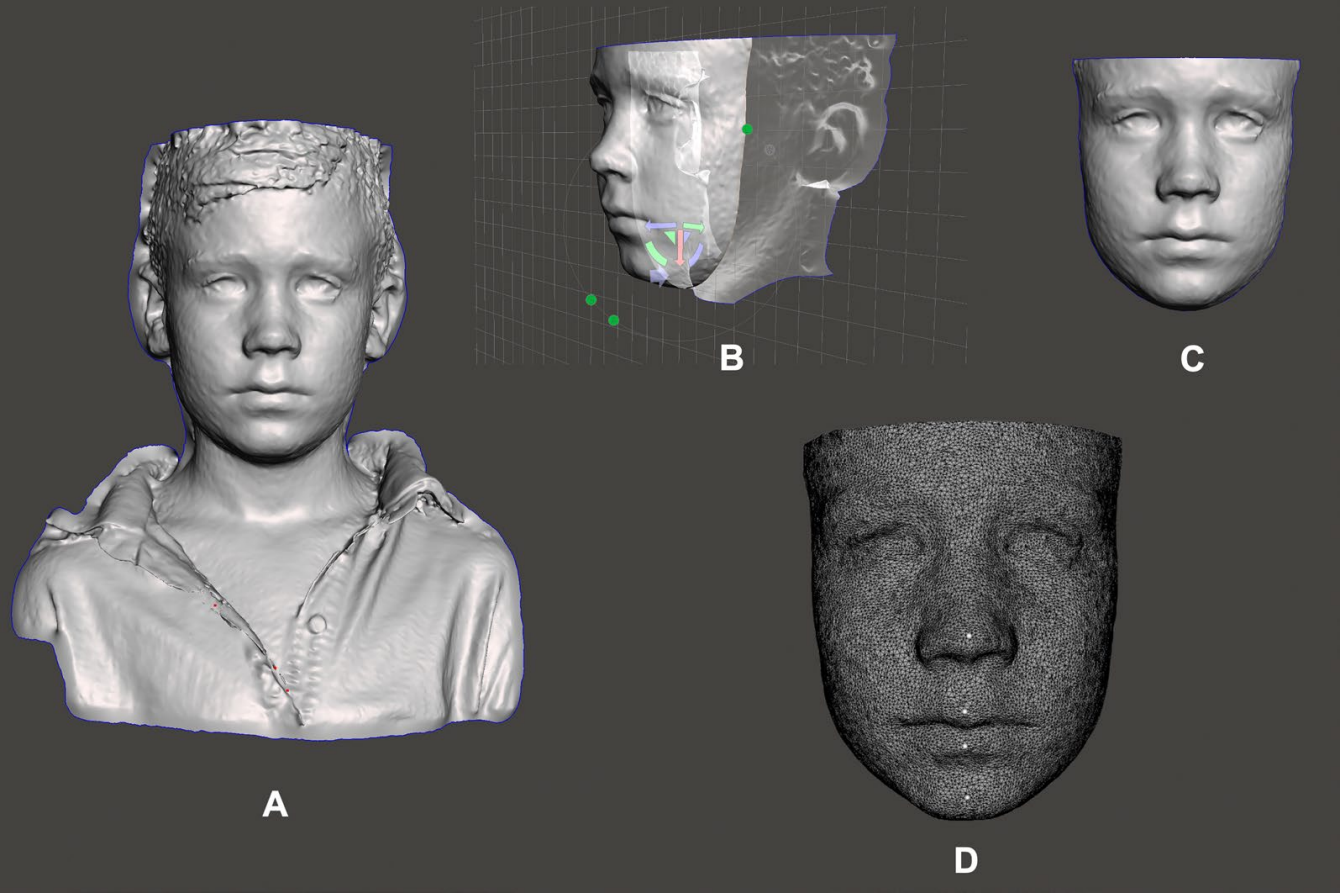
Preparation of the facial models for the 3D analysis. **A** Raw model, **B** cropping model; **C** cropped model; **D** landmark identification for quantitative point-to-point measurements

The midsagittal plane also was used as the reference when applying the “Transforms” tool of the software 3D Slicer (open-source, www.slicer.org). The Plane of Camper was set parallel to the XY plane, and the midsagittal plane was matched to the YZ plane. The control of roll was carried out in frontal view with right and left facial sides positioned symmetrically relative to the XZ plane. Models were trimmed using the “Easy clip” tool (Fig. 1 A-C). Automated computerized registration of the models was based on the Region of Interest (ROI) method [24] using the “Surface registration” tool.

**Fig. 2:**
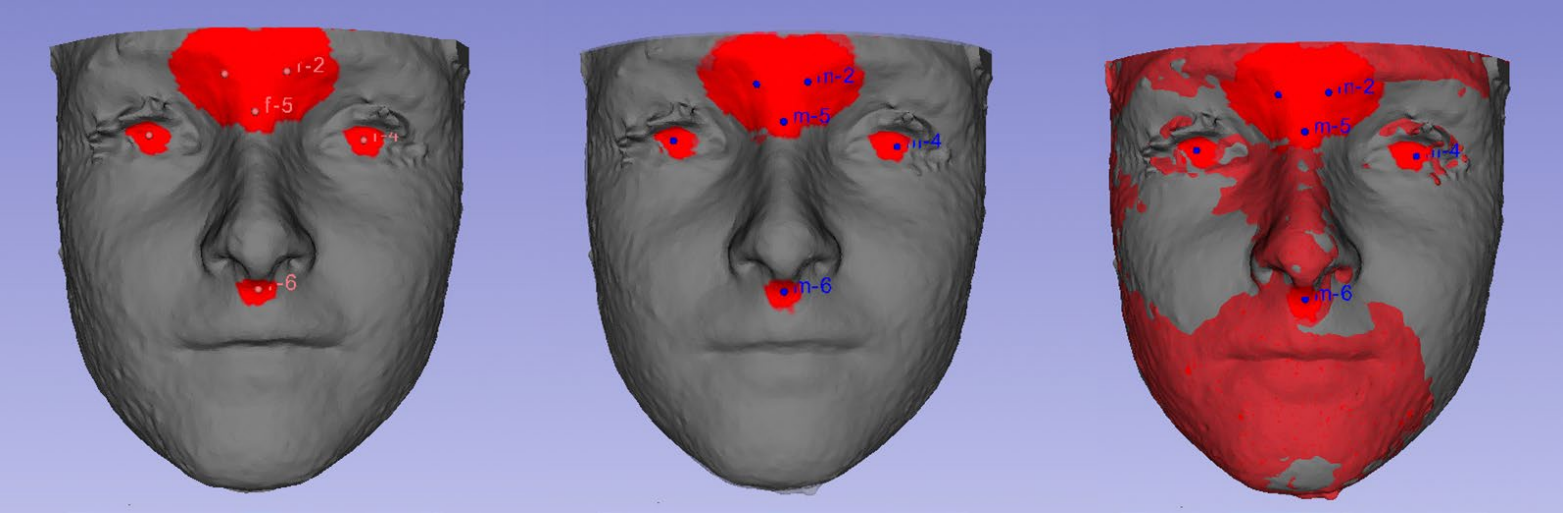
Registration of the T0 and T1 models using fiduciary landmarks

**Fig. 3:**
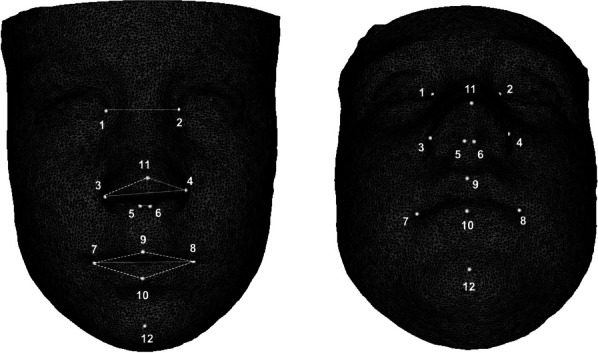
Landmarks and linear and angular measurements

After the selection of six landmarks (right and left center of the eyes, right and left mid-upper most concave contour of the orbits, glabella, and subnasale), the computer calculated a ROI of 5 triangles around the selected landmark and used the ROIs for the T0 and T1 automated superimposition (Fig. 2). Twelve landmarks were plotted on the models (Fig. 3) to allow quantitative point-to-point calculation of 3D linear and angular measurements (Fig. 3) as independent time points.

The landmarks identified on T0 and T1 3D images [14] are reported in Table 1 and Fig. 3.

**Table 1:** Landmark definitions

Point number	Point name	Point definition
1 and 2	Endocanthion	Point at the inner commissure of the eye fissure
3 and 4	Alar point	The most lateral point on each alar contour (adjusted on the frontal and lateral views)
5 and 6	Columella point	The point located at the lateral aspects of the central region of the columella (on the submental view)
7 and 8	Cheilion	The point located at each labial commissure
9	Labiale superius (Ls)	The midpoint of the vermilion line of the upper lip
10	Labiale inferius (Li)	The midpoint of the vermilion line of the lower lip
11	Pronasale (Prn)	The most anterior midpoint of the nasal tip (adjusted on the frontal and lateral views)
12	Soft tissue Pogonion	The most anterior point of the soft tissue of the chin

The definitions of the 3D linear and angular measurements are reported in Table 2 and Fig. 3.

**Table 2:** Definitions of 3D linear and angular measurements

Measurement	Definition
Intercanthal width (mm)	Linear distance between the endocanthion points (1 and 2)
Nasal width (mm)	Linear distance between the most lateral point on each alar contour (adjusted on the frontal and lateral views)
Nasal columella width (mm)	Linear distance between the points located at the lateral aspects of the central region of the columella (on the submental view)
Mouth width (mm)	Linear distance between the points located at each labial commissure
Nasal tip angle (degree)	Angle formed by AlarRight-Prn-AlarLeft
Upper lip angle (degree)	Angle formed by CheilionRight-Ls-CheilionLeft
Lower lip angle (degree)	Angle formed by CheilionRight-Li-CheilionLeft

Three-dimensional displacements between T0 and T1 were calculated on the projected *X* (right-left), *Y* (anterior–posterior), *Z* (superior-inferior) axes, and the 3D Euclidean distances of the nose, upper lip, lower lip, and chin (Fig. 1D). All 3D imaging procedures and measurements were performed by the same examiner (B.Q.S.), who was blinded on the type of expander used.

### Sample size

To calculate the sample size, nasal width was used as the primary outcome in the G*Power software (version 3.1, Heinrich-Heine-Universität Düsseldorf, Düsseldorf, Germany). Based on a previous study [16], 0.956 mm and 0.794 mm of standard deviation for each group was adopted. The ratio between individuals of both groups was 1:1. The accepted clinical difference between groups was 1 mm (effect size of 1.13), α equal to 0.05 and the power of 80%. Thus, a sample of 28 individuals (14 in the RME and 14 in the SME) was necessary to reject the null hypothesis of no difference between groups.

### Randomization and allocation concealment

A computer-generated random number list was used to allocate patients to treatments. Block randomization was used to assign the same number of patients to each treatment group.

The allocation sequence was concealed by the statistician (M.N.), who used opaque and sealed envelopes, sequentially numbered. The envelopes were opened by the operators only when the expander was prepared for cementation.

### Blinding

Patients and parents did not know which group (conventional RME or Leaf) they were assigned to, but they could not be blinded concerning the type of expander used because of the different modalities of activation of the screw.

Clinicians could not be blinded about the treatment that they were providing. The examiner (B.Q.S.) was blinded on the type of expander used.

### Statistical analysis

The intra-rater agreement was calculated on 10 repeated measures (after a 2-week washout period) with the intraclass correlation coefficients (ICCs). Descriptive statistics were performed using mean and standard deviation for quantitative variables and frequency and percentage for qualitative variables.

To assess the difference between treatments, linear and angular measurements were compared by means of analysis of covariance (ANCOVA) test using the value at T0 as a covariate.

The comparison between displacements of the nose, upper lip, lower lip, and pogonion between T0 and T1, evaluated in the three-dimensional perspective (*x*, right-left; *y*, anterior–posterior; *z*, superior–inferior; and 3D Euclidean distance) in the two treatment groups was assessed using the *t*-test.

For each statistical model, the estimate of the treatment effect (the estimate of the difference between the two treatments used), the *P*-value, and the 95 percentage confidence interval (CI) were provided.

Statistical analysis was carried out according to the intention-to-treat method. The unit of analysis was represented by the patient. All statistical computations were performed with statistical software (JMP vers. 13.0.0, SAS Institute Inc., Cary, NC, USA, and MedCalc version 19.6.4, MedCalc Software Ltd., Ostend, Belgium).

## Results

The ICC ranged from 0.50 (LL *x*) to 0.99 (Mouth width) between the two sets of measurements (Additional file 3: Table 1). According to Landis and Koch [25], the strength of agreement ranged from moderate to almost perfect.

Twenty-eight patients were enrolled in the trial and were allocated randomly to the maxillary expansion with the two types of screw. Fourteen patients were allocated to the conventional RME expander (RME group) and fourteen patients were allocated to the Leaf expander (SME group). The patients were recruited and treated in the Orthodontic Clinic of the Careggi University Hospital, Florence, Italy, from October 2016 to November 2018. The last 12-month follow-up was carried out in November 2019.

Baseline demographic and clinical characteristics of the patients are shown in Table 3. No significant differences between the two groups were observed for any of the variables at the beginning of therapy. All patients received the treatment assigned by randomization. There were no withdrawals from the trial and no deviations from the protocol.

**Table 3:** Baseline values in the two treatment groups

Variable	RME *N* = 14	SME *N* = 14	*P*-value *t*-test
Gender (F)	6 (43%)	6 (43%)	1.000*
Age (years)	8.2 (1.3)	7.9 (0.8)	0.475
Intercanthal width (mm)	33.6 (7.0)	31.6 (2.0)	0.312
Nasal width (mm)	29.9 (3.1)	28.9 (1.8)	0.325
Nasal columella width (mm)	5.9 (1.0)	6.5 (0.7)	0.097
Mouth width (mm)	38.0 (4.2)	36.5 (2.8)	0.273
Nasal tip angle	150.8 (14.4)	149.7 (22.4)	0.876
Upper lip angle	150.1 (12.3)	143.7 (11.5)	0.167
Lower lip angle	148.3 (9.9)	149.8 (8.6)	0.667

Standard deviation in parentheses

*Fisher exact test

Duration of active therapy was 1.0 ± 0.4 months in the RME group, and it was 4.5 ± 1.1 months in the SME group (difference 3.5 months, *P* < 0.0001).

In Table 4, descriptive statistics and statistical comparisons for T1-T0 differences are illustrated.

**Table 4:** Linear (3D Euclidean distance) and angular differences between T0 and T1 in the two treatment groups

Variable	RME *N* = 14	SME *N* = 14	Adjusted difference	95%CI	*P*-value ANCOVA
Intercanthal width (mm)	0.7 (0.9)	0.2 (0.8)	0.7	0.0; 1.3	0.044
Nasal width (mm)	1.7 (1.0)	0.3 (1.2)	1.3	0.4; 2.2	0.005
Nasal columella width (mm)	0.4 (1.3)	− 0.0 (1.0)	0.1	− 0.8;1.0	0.799
Mouth width (mm)	0.5 (2.0)	1.1 (2.8)	− 0.4	− 2.4; 1.5	0.647
Nasal tip angle	− 0.3 (11.2)	2.4 (16.0)	− 2.1	− 10.8; 6.5	0.617
Upper lip angle	− 2.5 (11.5)	0.4 (7.5)	− 1.8	− 9.7; 6.0	0.633
Lower lip angle	0.6 (8.8)	2.2 (4.9)	− 2.1	− 7.2; 3.0	0.395

Standard deviation in parentheses

The primary outcome variable “nasal width” showed a statistically significant difference between the two groups (1.3 mm larger in the RME group, 95% CI from 0.4 to 2.2, *P* = 0.005). Also, intercanthal width showed a difference between treatments (0.7 mm larger in the RME group, 95% CI from 0.0 to 1.3, *P* = 0.044). Nasal columella width, mouth width, nasal tip angle, upper lip angle, and lower lip angle did not show any statistically significant differences.

The displacements of the nose, upper lip, lower lip, and pogonion between T0 and T1 evaluated in the three-dimensional perspective in the two treatment groups are reported in Table 5. The distances of the *Y* (anteroposterior) components of the nasal landmark showed a slight but statistically significant difference between the two groups (0.5 mm in favor of forward displacement of the RME group, *P* = 0.040). Also, the distance of the *Z* (superior–inferior) components of the lower lip landmarks was statistically significant (0.9 mm in favor of downward displacement of RME group, *P* = 0.027). All the other comparisons of the assessment of the distances were not statistically significant.

**Table 5:** Displacements of the nose, upper lip, lower lip, and pogonion between T0 and T1 evaluated in the three-dimensional perspective in the two treatment groups

Variable	RME *N* = 14	SME *N* = 14	Adjusted difference	95%CI	*P*-value *t*-test
Nose 3D	2.1 (1.0)	1.8 (1.3)	0.3	− 0.6; 1.2	0.516
Nose *x*	0.7 (0.9)	0.7 (0.5)	0.0	− 0.6; 0.6	0.991
Nose *y*	1.0 (0.7)	0.5 (0.4)	0.5	0.0; 1.0	0.040
Nose *z*	1.4 (0.8)	1.3 (1.5)	0.1	− 0.9; 1.0	0.878
UL 3D	2.5 (1.7)	1.8 (1.0)	0.7	− 0.4; 1.7	0.215
UL *x*	0.9 (0.6)	0.9 (0.8)	0.0	− 0.6; 0.5	0.916
UL *y*	1.4 (1.2)	0.7 (0.5)	0.7	− 0.1; 1.4	0.077
UL *z*	1.5 (1.6)	1.2 (0.9)	0.3	− 0.7; 1.3	0.539
LL 3D	2.9 (1.3)	2.2 (1.1)	0.7	− 0.2; 1.7	0.119
LL *x*	1.0 (0.6)	0.8 (0.6)	0.2	− 0.3; 0.6	0.498
LL *y*	1.3 (1.3)	1.4 (0.9)	− 0.1	− 0.9; 0.8	0.897
LL *z*	2.1 (1.1)	1.2 (1.3)	0.9	0.1; 1.7	0.027
Pg 3D	3.3 (1.5)	3.5 (1.7)	− 0.2	− 1.4; 1.0	0.746
Pg *x*	1.2 (1.1)	1.4 (1.2)	− 0.2	− 1.1; 0.7	0.663
Pg *y*	1.7 (1.2)	2.1 (1.6)	− 0.4	− 1.5; 0.7	0.478
Pg *z*	2.1 (1.5)	2.0 (1.1)	0.1	− 0.9; 1.1	0.840

Standard deviation in parentheses

*x*, right–left; *y*, anterior–posterior; *z*, superior–inferior; 3D, Euclidean 3D distance, UL, upper lip; LL, lower lip; Pg, pogonion

## Discussion

The objective of this RCT was to compare the facial soft tissue effects produced by two different types of maxillary expanders, the conventional rapid maxillary expander, and the slow maxillary expander (Leaf expander), the treatment effects of which were evaluated on facial 3D images obtained by digital stereophotogrammetry. In particular, the main hypothesis was that there were not statistically significant differences in soft tissue changes in the nasal area. To our knowledge, this is the first RCT that compared the facial soft tissues changes produced by RME versus SME.

The primary outcomes of our study were the short-term effects of RME versus SME on the soft tissue of the nasal area evaluated by comparing the difference between the intercanthal width, nasal width, nasal columella width, and nasal tip angle, given the close anatomical relationship between the maxilla and the nasal area. The only variables that showed significantly greater increases in the RME group were intercanthal width, nasal width, and the displacement of the nasal landmark on the *y*-axis (0.7 mm, 1.3 mm, and 0.5 mm, respectively). These differences, though statistically significant, probably are clinically insignificant, if we consider as a threshold for a clinically significant change a value of 1.0–1.5 mm. Similar conclusions were drawn by Johnson et al. [26] who found that the effects of RME on nasal width indicated that the actual amount of change was less than 1.5 mm, an increase that was not considered as clinically significant. Additionally, Akhayer et al. [14] reported an increase in nasal width of 1.02 mm, a finding that was statistically significant but not clinically relevant, as it was less than 2 mm. It should be noted, however, that both these studies [14, 26] did not include comparisons with a control group. A limitation of all these studies (included the present RCT) is their short-term nature. It is not clear if the increase in nasal width will be stable in the long term. Studies with longer follow-ups, therefore, are needed to clarify this aspect.

The other analyzed variables of the nasal area (nasal tip angle and nasal columella width) did not show any statistically significant differences between the two groups. A direct comparison with the literature is difficult because previous studies that used 3D stereophotogrammetry evaluated the effects on the facial soft tissues produced by RME alone or in comparison with untreated subjects.

Baysal et al. [16] analyzing the hard and soft tissues difference between a group of patients treated with RME and an untreated control group, found that the only variable that showed a statistically significant difference was nasal width (alar base width), which was approximately 1 mm greater in the RME group than in the controls. Similarly, Fastuca et al. [27] found a significant increase of about 2 mm in nasal width in a sample treated with RME with respect to untreated controls. These findings are similar to the one reported in the current study, though the control sample in our study consisted of patients treated with SME.

As for intercanthal width, Baysal et al. [25] reported no statistically significant difference (0.4 mm) between the RME group and the untreated controls. In the present study, RME produced a significant increase in intercanthal width respect to SME though the amount of the difference was similar (0.7 mm).

Regarding the area of the mouth, Baysal et al. [25] and Fastuca et al. [27] reported no significant changes in mouth width in patients treated with RME with respect to untreated controls (0.6 and 0.8 mm, respectively). This finding agrees with the current study that showed no significant difference between RME and SME (− 0.4 mm).

In our study, the RME produced a slight but statistically significant greater forward displacement of the tip of the nose and downward displacement of the lower lip with respect to SME (0.5 mm and 0.9 mm, respectively). These findings can be explained, at least in part, by a forward and downward displacement of the maxilla following RME [28].

A limitation of this study is that only one center of the original two-center study collected 3D images, so it was not possible to investigate any differences between the 2 centers. Another limitation was that for a few variables (UL *x*, LL *x*, Pg *y*, and Pg *z*) the ICC values were between 0.50 and 0.60 indicating moderate agreement [25]. Finally, the results of this short-term RCT should be re-evaluated at a long-term follow-up.

## Conclusions

RME produced significant facial soft tissue changes when compared to SME. Specifically, RME induced greater increases in both nasal and intercanthal widths (1.3 mm and 0.7 mm, respectively). RME produced also greater forward displacement of the tip of the nose and downward displacement of the lower lip with respect to SME (0.5 mm and 0.9 mm, respectively). These findings, though statistically significant are probably not clinically relevant.

### Supplementary Information

**Additional file 1: Fig. 1.** Conventional rapid maxillary expander.

**Additional file 2: Fig. 2.** Leaf expander

**Additional file 3: Table 1.** Intra-rater agreement with intraclass correlation coefficient (ICC) and error calculated with Dahlberg’s formula.

## Data Availability

The data underlying this article will be shared to the corresponding author after reasonable request.
