# Supplementary figures and images for: Comparison of the effects on facial soft tissues produced by rapid and slow maxillary expansion using stereophotogrammetry: a randomized clinical trial
Source: Prog Orthod. 2024 Jan 3;25:1. doi: 10.1186/s40510-023-00498-9 (PMC10761642; doi:10.1186/s40510-023-00498-9)

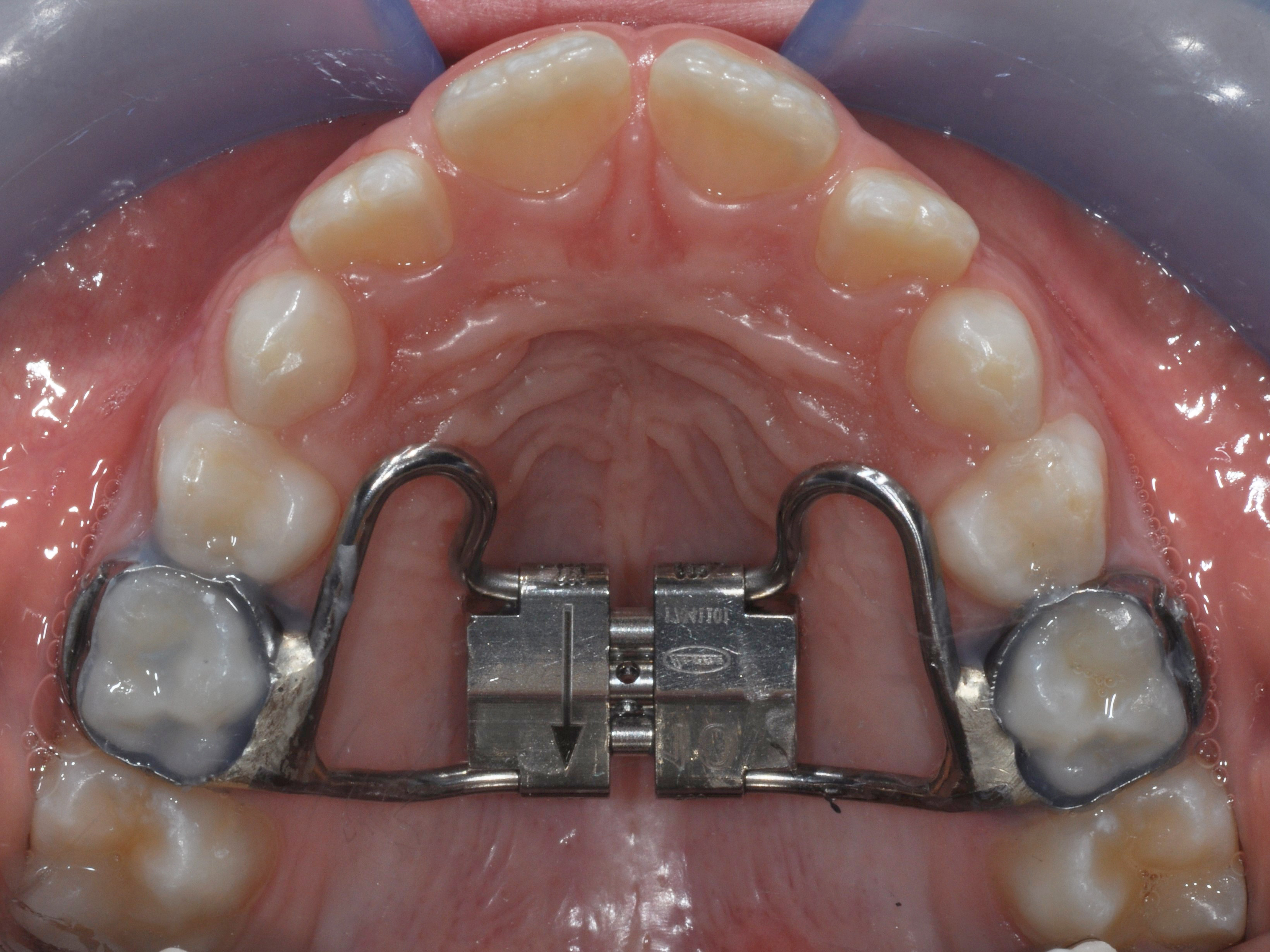

Supplement: Supplementary file 1 — Additional file 1: Fig. 1. Conventional rapid maxillary expander. [file 40510_2023_498_MOESM1_ESM.jpg]

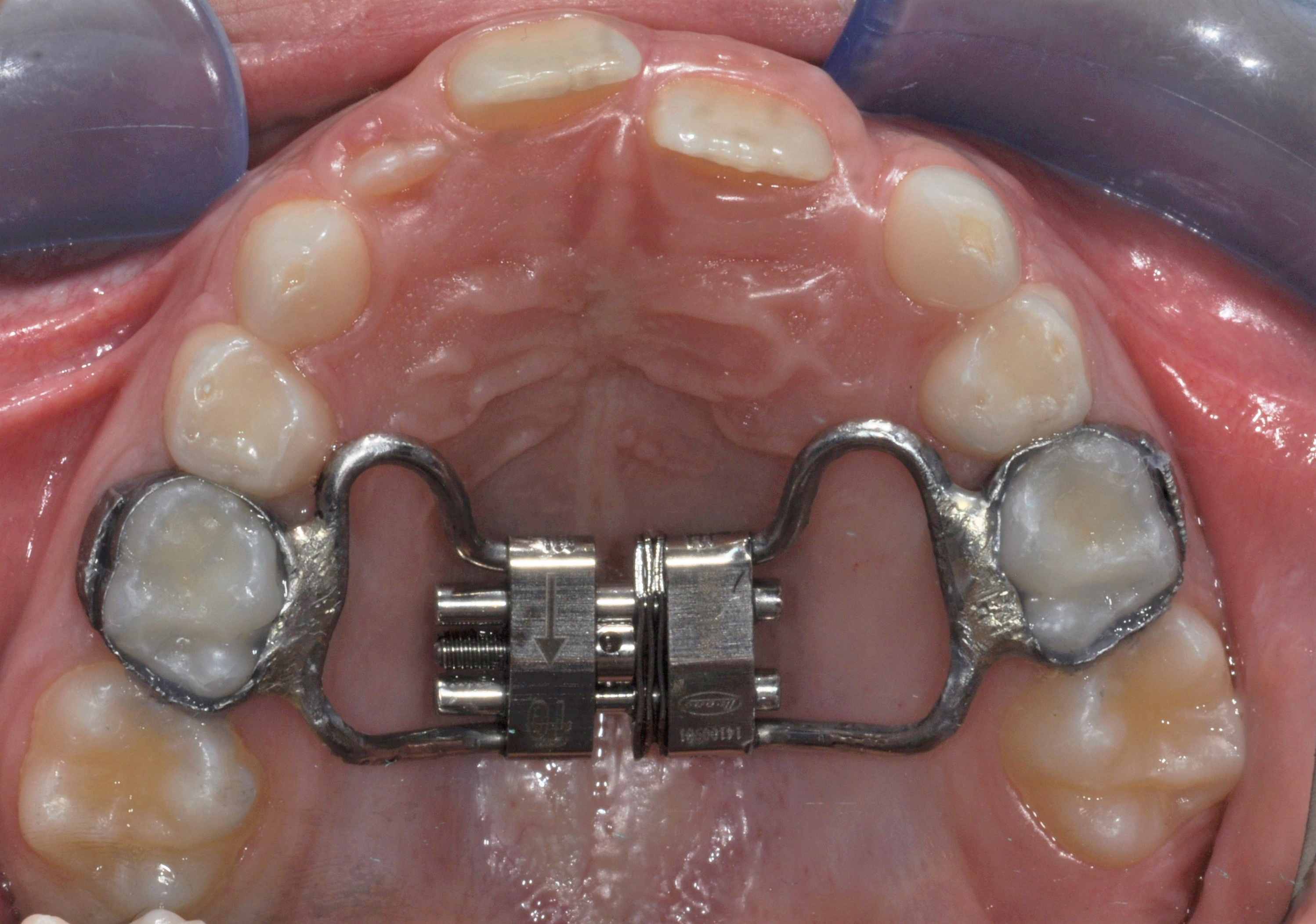

Supplement: Supplementary file 2 — Additional file 2: Fig. 2. Leaf expander [file 40510_2023_498_MOESM2_ESM.jpg]
