# Supplementary material for: Comparison of the effects on facial soft tissues produced by rapid and slow maxillary expansion using stereophotogrammetry: a randomized clinical trial
Source: Prog Orthod. 2024 Jan 3;25:1. doi: 10.1186/s40510-023-00498-9 (PMC10761642; doi:10.1186/s40510-023-00498-9)
Supplement: Supplementary file 3 — Additional file 3: Table 1. Intra-rater agreement with intraclass correlation coefficient (ICC) and error calculated with Dahlberg’s formula. [file 40510_2023_498_MOESM3_ESM.docx]

Supplementary Table 1: Intra-rater agreement with Intraclass correlation coefficient (ICC) and error calculated with Dahlberg’s formula.

| Variable | ICC | 95%CI | Error (mm) |
| --- | --- | --- | --- |
| Intercanthal width | 0.84 | 0.52; 0.96 | 0.7 |
| Nasal width | 0.98 | 0.89; 0.99 | 0.5 |
| Nasal columella width | 0.98 | 0.91; 0.99 | 0.1 |
| Mouth width | 0.99 | 0.97; 1.0 | 0.4 |
| Nasal tip angle | 0.63 | 0.10; 0.89 | 7.8* |
| UL angle | 0.92 | 0.73; 0.98 | 2.9* |
| LL angle | 0.67 | 0.16; 0.90 | 5.8* |
| Nose 3d | 0.80 | 0.38; 0.95 | 0.6 |
| Nose x | 0.69 | 0.12; 0.91 | 0.5 |
| Nose y | 0.69 | 0.19; 0.91 | 0.4 |
| Nose z | 0.85 | 0.49; 0.96 | 0.5 |
| UL 3D | 0.73 | 0.23; 0.93 | 0.7 |
| UL x | 0.52 | -0.07; 0.85 | 0.5 |
| UL y | 0.67 | 0.16; 0.90 | 0.7 |
| UL z | 0.73 | 0.22; 0.93 | 0.8 |
| LL 3D | 0.79 | 0.38; 0.94 | 0.5 |
| LL x | 0.50 | -0.15; 0.85 | 0.5 |
| LL y | 0.88 | 0.54; 0.97 | 0.4 |
| LL z | 0.67 | 0.10; 0.91 | 0.7 |
| Pg 3D | 0.66 | 0.15; 0.90 | 0.9 |
| Pg x | 0.80 | 0.21; 0.95 | 0.6 |
| Pg y | 0.51 | -0.04; 0.85 | 1.2 |
| Pg z | 0.59 | -0.07; 0.88 | 0.8 |

X – right-left; Y – anterior-posterior; z – superior-inferior; 3D – Euclidean 3D distance

UL – upper lip; LL – lower lip; Pg – pogonion; * = degrees
